# Effect of Plant-Derived and Microbial Feed Additives on the Growth Performance and Biochemical Composition of Juvenile Sea Cucumber *Stichopus monotuberculatus*

**DOI:** 10.1155/anu/6521606

**Published:** 2025-03-21

**Authors:** Jiayu Zhang, Chao Li, Yan Zhao, Yanchao Chai, Haiqing Wang

**Affiliations:** ^1^School of Marine Biology and Fisheries, Hainan University, 58 People Road, Haikou 570228, China; ^2^Qingdao Institute of Bioenergy and Bioprocess Technology, Chinese Academy of Sciences, No. 189 Songling Road, Qingdao 266101, China; ^3^Marine Science and Engineering College, Nanjing Normal University, 1 Wenyuan Road, Nanjing 210023, China

**Keywords:** biochemical composition, enzyme activity, feed additives, growth performance, *Stichopus monotuberculatus*

## Abstract

Low growth and survival rates (SRs) are common challenges confronted in cultivation of sea cucumbers, particularly during juvenile stage. Given the significance of feed components in aquaculture, it is essential to explore various additives in formulated feed for juvenile sea cucumber *Stichopus monotuberculatus*. In this study, juveniles were fed a basal diet supplemented with 500 mg/kg of tea powder, 100 mg/kg of allicin, 20 mL/kg of probiotics, and 100 mL/kg of earthworm hydrolysate (EH) over a 56-day feeding trial. The SRs, growth performance, nonspecific immunity, antioxidant activity, nutrient composition, and digestive activity of juveniles were evaluated. The results showed that all four feed additives positively affected the digestive ability of *S. monotuberculatus*. Tea powder, allicin, and probiotics in the diet significantly enhanced the growth performance of the juveniles, while EH exhibited a beneficial impact on the nutrient accumulation. Additionally, tea powder and allicin were found to enhance immune responses. Therefore, this study provided insights into how feed additives affect growth, digestibility, and immune responses in aquatic animals, offering valuable information for developing effective dietary strategies for tropical sea cucumbers.

## 1. Introduction

Sea cucumbers are widely distributed in oceans worldwide. They play a crucial role in maintaining marine ecological balance, regulating carbonate budgets, and interacting with microbes in the sediments [1–3]. Moreover, sea cucumbers are considered an economically important farmed echinoderm species in China due to their abundance of minerals and bioactive compounds that benefit human health [4]. *Stichopus monotuberculatus*, a prominent species in coral reef ecosystems, is highly valued for its nutritional and medicinal properties [5, 6]. The growing demand for sea cucumbers has driven increased investments in commercial farming [7–9]. However, juvenile sea cucumbers exhibit slow growth rate (GR) and allometric growth patterns, which limit the efficiency of sea cucumber farming [10].

Current study on sea cucumber feed primarily focuses on temperate species, while tropical species remain largely overlooked. This gap in research impedes the advancement of sustainable aquaculture practices [11]. Marine organisms in temperate regions generally require higher energy levels for growth and metabolism due to lower temperatures compared to tropical species [12]. This suggests that existing formulated feeds may be unsuitable for tropical species. Additionally, a study on Pacific red snapper (*Lutjanus peru*) found that while elevated levels of docosahexaenoic acid (DHA) are crucial for cold-water species, they may present challenges for warm-water species [13]. Therefore, it is urgent to develop appropriate feed for tropical sea cucumber aquaculture.

Feed additives are substances, such as molecules, compounds, or organisms, which were added to animal feeds to enhance its nutritional value, improve growth, or achieve other benefits [14]. Various plant-derived additives [15], animal-derived supplements [16], and probiotics [17, 18] have been used in the diet of sea cucumbers. Therefore, identifying optimal feed additives is expected to improve feed quality and palatability for juvenile sea cucumbers, thereby, stimulating growth, enhancing immunity, and reducing mortality rates.

Partridge tea (*Mallotus obongifolius*), also traditionally known as “glossy ganoderma,” has significant medicinal and economic value. Predominantly found in Hainan Province, China, it is commonly used as a substitute tea beverage with strong local characteristics [19, 20]. Tea polyphenols are primary active components, offering a rich natural source of antioxidants [21]. Research has demonstrated that tea polyphenols significantly enhance the growth, antioxidant capacity, and immune response of aquatic organisms, highlighting their importance in aquaculture [22–24]. Allicin (C_6_H_10_OS_2_), a naturally occurring compound rich in bioactive substances, is widely used in aquaculture for its effectiveness in disease prevention and product quality improvement [25]. Earthworms, with their high protein content and diverse amino acid profile, are a promising source of animal protein due to their ease of cultivation [26]. Earthworm hydrolysate (EH), a nutrient-rich additive, contains a higher concentration of bioactive compounds and vitamins compared to earthworm powder [27]. Effective microorganisms (EMs), well-studied probiotics, have been shown to enhance GRs, immunity, and disease resistance in various aquatic species [28]. The probiotics used in this study primarily consist of lactic acid bacteria, yeast, and photosynthetic bacteria.

This study aimed to identify the optimal feed additives for trophic juvenile sea cucumbers and understand how these specific additives affect their growth, digestive efficiency, and immunity. The effects of four additives (partridge tea, allicin, probiotics, and EH) were evaluated on the growth performance, nutritional composition, nonspecific immunity, and digestive enzyme activity of juvenile *S. monotuberculatus*. This information may serve as a guide for cultivating other tropical sea cucumber species and support the sustainable development of the tropical sea cucumber industry.

## 2. Materials and Methods

### 2.1. Experimental Diets

The foundation of the diets in this study was derived from those utilized in sea cucumber farming. These basic diets consisted of *Sargassum denticarpum* meal, *Sargassum thunbergii* meal, formula feed, and sea mud, combined in equal proportions of 1:1:1:1. The basal diets used in this study were derived from those utilized in sea cucumber aquaculture and were modified based on relevant researches [29, 30]. The proximate composition of mixed basal feed included 13.69% crude protein, 1.92% crude fat, and 7.31% ash (Table 1). The mixture was powdered and sieved through a 100-mesh screen. All the feed ingredients were procured from Qingdao Haifute Ecological Science and Technology Company.

The study used four dietary additives: partridge tea powder, allicin, probiotics, and EH. Partridge tea, purchased from a market in Haikou, was processed into powder by drying, grinding, and sieving through a 60-mesh screen. Allicin was obtained from Mukun Fish Medicine Co. Ltd. and probiotics from Guangzhou Liyang Aquatic Science and Technology Company. The EH originated from a vermiculture facility in Hainan. The earthworms were homogenized and fermented with EM bacteria to break down macromolecular proteins into free amino acids. The crude composition of partridge tea powder, allicin, probiotics, and liquid EH is presented in Table 2.

There were five treatments: Control (no additives), Tea group (500 mg/kg partridge tea), Allicin group (100 mg/kg allicin), Probiotics group (20 mL/kg probiotics) and EH groups (100 mL/kg EH; Table 3). All treatments comprised a basal diet, either with or without additives. For partridge tea powder and allicin, the optimal additive amounts were established to be 500 and 100 mg/kg, respectively, based on findings from the pre-experiment and relevant literature [31, 32]. A 2-week pre-experiment was conducted to determine the optimal concentration of the probiotic and EH. The probiotic concentration was established at five levels: 0, 5, 10, 20, and 30 mL/kg. The concentration of the EH was set at four levels: 20, 50, 100, and 150 mL/kg. Concentrations were determined based on relevant studies and then optimized according to their findings [27]. After 2 weeks of culture, the optimal level of probiotics and EH were determined to be 20 and 100 mL/kg, respectively, based on the growth performance of sea cucumbers in this experiment. The decision was made based on a comprehensive analysis of survival rates (SRs) and GRs.

### 2.2. Sea Cucumber Husbandry and Experimental Design

Juvenile *S. monotuberculatus* sea cucumbers were obtained from Kuntian Marine Bio-technology Co., Ltd. in Wenchang, Hainan, China. Prior to the experiments, they underwent disinfection with a 10% florfenicol solution at a dosage of 0.5 mL/m^3^, then were acclimatized to the laboratory conditions in a 300 L tank for 14 days. Throughout the acclimation period, the sea cucumbers were fed with basic diets daily.

A total of over 300 juvenile sea cucumbers with similar sizes were selected for the experiment. Twenty sea cucumbers were allocated randomly into each of the triplicate tanks (30 cm × 20 cm × 17 cm; *n* = 5 diet treatments, *n* = 15 tanks) with an average weight of 2.33 ± 0.24 g. Throughout the 56-day feeding trial, sea cucumbers were fed daily at 4:00 p.m. with an amount of approximately 5% of their total wet weight in each tank. The feeding quantity was adjusted according to the feeding status and body weight growth of the juveniles to ensure that they were slightly overfed. Residuals were collected at 2:00 p.m. the next day, and the feeding amount was determined.

The culture containers were aerated, and 1/3 to 1/2 of the seawater volume was replaced every day. Residuals and feces were removed by siphoning before water change. Temperature, salinity and pH were measured every 2 days, with salinity maintained around 30‰ and pH at approximately 8.0. The room temperature was kept at 25°C, while the water temperature was 25 ± 1°C.

### 2.3. Growth Performance and Feeding Rate

The body weight of sea cucumbers was measured on days 0, 14, 28, 42, and 56 throughout the experiment. Residual feed was gathered daily, dried, and weighed to determine the feeding quantity. SR, GR, specific GR (SGR), ingestion rate (IR), and feed conversion ratio (FCR) of sea cucumbers in each treatment were calculated as follows:

  $$\begin{array}{c} \text{SR}=\frac{N_{t}}{N_{0}}\times 100\%, \end{array}$$

  $$\begin{array}{c} \text{GR}=\frac{W_{t}-W_{0}}{W_{0}}\times 100\%, \end{array}$$

  $$\begin{array}{c} \text{SGR}=\frac{\ln W_{t}-\ln W_{0}}{T}\times 100\%, \end{array}$$

  $$\begin{array}{c} \text{IR}=\frac{C}{\left[T\times \left(W_{0}+W_{t}\right)/2\right]}, \end{array}$$

  $$\begin{array}{c} \text{FCR}=\frac{C}{W_{t}-W_{0}}, \end{array}$$

where *W*_0_ and *W*_t_ were the initial and final wet body weight (g) of sea cucumbers, respectively. *N*_0_ and *N*_t_ were the number of sea cucumbers at the start and the end of the experiment. *T* was the duration of the experiment (days); *C* was food intake (g).

### 2.4. Biochemical Analysis

The body wall tissues of sea cucumbers were sampled, and 1 g of the sample was freeze-dried and then ground to determine their nutritional composition. The moisture content was assessed by drying to a constant weight at 105°C. Ash content was determined through incineration at 550°C for 5 h using a muffle furnace.

Crude protein was determined and analyzed using a Dumas Nitrogen Determinator (Rapid Maxn Exceed System, Elementar, Germany). Crude lipid was measured by Soxhlet extraction method (GB/T 6433-1994) with petroleum ether with a boiling point of 30–60°C (Fat Analyzer, ANKOM XT15i Extractor, USA).

The amino acid composition was determined using HPLC (Waters e2695+2998PDA, USA). Approximately 0.5 g of body wall tissue was placed in a 10 mL glass ampule, followed by the addition of 2.5 mL of 0.1 M HCl. The mixture was then cooled on ice, sonicated for 20 min, and subsequently centrifuged at 4°C and 10,000 rpm for 10 min. Following this, 300 μL of supernatant was extracted and 75 μL of 0.1 M PITC–acetonitrile solution was added. The resulting solution was vortexed for 15 s, allowed to stand for 2 min, then centrifuged, and the bottom layer was collected for analysis.

Fatty acids were analyzed with GC-MS (Agilent Technologies 5977-7890 GCMS, USA). Post lipid extraction, the fatty acids were esterified to fatty acid methyl esters through petroleum ether–methanol for 1 h. Subsequently, 1 mL of distilled water was added and the mixture was centrifuged at 6000 rpm for 10 min. The supernatant was then extracted, filtered through a 0.22 μm filter, and prepared for analysis.

### 2.5. Digestive and Antioxidant Enzyme Activity

In the final phase of the experiment, 10–15 juvenile sea cucumbers were randomly selected from each treatment group. The body wall surface was delicately wiped and promptly weighed. Subsequently, the juveniles were cooled on ice and dissected along the midline of the abdomen with scissors. The celomic fluid was meticulously extracted and placed in precooled centrifuge tubes and maintained under icy conditions to facilitate immune-related enzyme activity assessment within 12 h. Immune function markers in the celomic fluid, such as superoxide dismutase (SOD), catalase (CAT), total antioxidant capacity (T-AOC), and lysozyme (LZM), were analyzed using assay kits from Nanjing Jiancheng Bioengineering Institute, China.

Simultaneously with the collection of celomic fluid, the intestines of 10–15 sea cucumbers were flash-frozen in liquid nitrogen for subsequent analysis. The determination of lipase activity, pepsin, and *α*-amylase activity in the intestines was conducted. Protein concentration and lipase activity were assessed using the assay kit (Nanjing Jiancheng Bioengineering Institute, China), while the pepsin and *α*-amylase activities were examined with the assay kit from Solarbio Science and Technology (Beijing) Co. Ltd.

### 2.6. Statistical Analysis

The data measured were expressed as mean ± SEM (standard error). SPSS 25.0 software was used for statistical analysis. One-way ANOVA followed by LSD and Duncan's multiple range test was carried out to compare significant differences between treatments. Principal component analysis (PCA) was conducted to represent amino acids and fatty acids composition among the treatments. Before PCA analyses, percentage data were transformed with the equation: transfer data = 180/π × sin^−1^ ($\sqrt{\text{data}/100}$). A level of *p*  < 0.05 was considered significant.

## 3. Result

### 3.1. Effects of Different Additive Groups on Growth Performance

The results indicated that there was no significant difference in the SR among treatments with the highest rate in Tea group (96.67% ± 5.77%). The GR of sea cucumbers was significantly higher when they were fed diets supplemented with partridge tea powder compared to the Control group, the Probiotics group, and the EH group (*p*  < 0.05). The juvenile sea cucumbers fed EH exhibited the lowest GR (*p*  < 0.05). A comparison of GRs under different stages revealed that the maximum growth occurred in the group fed partridge tea powder from days 0 to 14. The GR during this period was 43.15%, significantly higher than in the other treatment groups and the Control group (*p*  < 0.05). The GR of juveniles fed with allicin exceeded that of the Control group overall time (Table 4).

**Figure 1:**
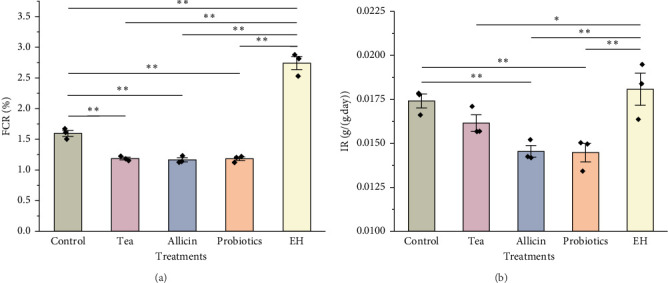
Feeding performance under different treatments. (a) Feed conversion ratio (FCR) and (b) ingestion rate (IR). Means with asterisks indicated significant differences among treatments (*⁣*^*∗*^*p*  < 0.05; *⁣*^*∗∗*^*p*  < 0.01). ◆ represent the means for each replicate under one treatment. EH, earthworm hydrolysate.

During the experiment, the FCR of sea cucumbers fed with partridge tea powder, allicin, and probiotics significantly decreased (*p*  < 0.01) compared to the Control group, with the Allicin group displaying the lowest ratio. Conversely, FCR in the EH group was markedly higher than the Control group (*p*  < 0.01). The daily IR of sea cucumbers fed with partridge tea powder, allicin, and probiotics was lower than that of the Control group, significantly under the Allicin and Probiotics groups, while the IR in the EH group was notably higher (*p*  < 0.01) compared to the Control group (Figure 1).

### 3.2. Effects of Different Dietary Additives on Nutritional Composition

The results revealed that the levels of crude protein in juvenile sea cucumbers across all groups ranged from 50.98% to 54.08%, and the protein level in the EH group was significantly higher compared to the other groups. The crude lipid content was from 1.92% to 2.47%, with a significantly higher level in EH group and a lower level in Allicin group (*p*  < 0.05). There were no significant differences observed in the levels of ash and moisture among the groups (Table 5).

The amino acid composition and contents are presented in Table 6. The essential amino acids (EAAs) contents in the tissue of *S. monotuberculatus* were relatively high, ranging from 42.19% to 43.68%. The three most abundant amino acids were glutamate (9.61–11.68 g/kg), glycine (7.11–7.98 g/kg), and aspartic acid (5.86–7.12 g/kg). No significant differences were found in amino acid levels among the Tea group, Probiotics group, and the Control group. However, the Allicin group had a significantly higher proportion of EAAs compared to the Control group. Additionally, the EH group exhibited elevated levels of histidine, valine, isoleucine, leucine, phenylalanine, lysine, and total EAAs compared to the Control group (*p*  < 0.05).

**Figure 2:**
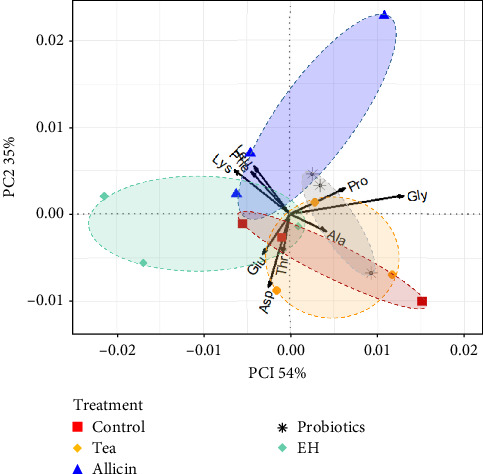
Principal component analysis (PCA) of amino acids (% of the total amino acids (TAAs)) in juvenile sea cucumber *Stichopus monotuberculatus* under different feed additives. Data was arcsine transformed. Ala, alanine; Arg, arginine; Asp, aspartic acid/asparagine; Glu, glutamic acid/glutamine; Gly, glycine; His, histidine; Ile, isoleucine; Leu, leucine; Lys, lysine; Met, methionine; Phe, phenylalanine; Pro, proline; Ser, serine; Thr, threonine; Tyr, tyrosine; Val, valine.

In terms of amino acids, the percentages of variance explained by PC1 were 54% with a high weight of Gly and Lys. The PC2 explained 35% of the total variance and was strongly influenced by Asp, Gly, and Glu. PC1 and PC2 generated accounted for 89% of the total variability (Figure 2). The amino acid composition and content in the Allicin group were distinctly different from those in the Control group, Tea group, and Probiotics group. In addition, the samples from Probiotics group and EH group were distinguished without any overlaps, indicating that there were differences in their effects on amino acid content. Whereas, the high degree of similarity between the Control, Tea group, and Probiotics group indicated that for the selected amino acid index, there was no significant effect between these treatments.

Fatty acid test results are shown in Table 7. The content of saturated fatty acids (SFAs) in the body wall of *S. monotuberculatus* ranged from 201.38 to 240.79 mg/kg, while unsaturated fatty acids (UFAs) ranged from 195.65 to 273.60 mg/kg. Hexadecanoic acid (C16:0), octadecanoic acid (C18:0), and eicosapentaenoic acid (C20:5) were the top three fatty acids in the body wall tissues. Comparatively, the Allicin group exhibited lower fatty acid content than the Control group, notably in eicosanoic acid (C20:0) and eicosapentaenoic acid (C20:2). Conversely, the EH group displayed higher fatty acid content compared to the Control group, particularly with eicosanoic acid (C22:0) at a significantly elevated level (*p*  < 0.05).

**Figure 3:**
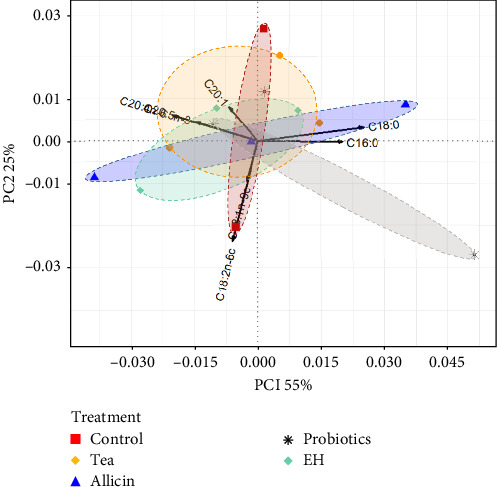
Principal component analysis (PCA) of fatty acids (% of the total fatty acids) in juvenile sea cucumber *Stichopus monotuberculatus* with different dietary additives. Data was arcsine transformed.

In terms of fatty acids, the PC1 explained 55% of the total variance and was strongly influenced by C18:0, C16:0 and C20:4*n−*6. The contribution of the PC2 was 25% with a high weight of C18:2*n*−6c and C18:1*n*−9c. The cumulative contribution of PC1 and PC2 was 80%. Among the fatty acids shown in Figure 3, the differences between the treatment groups and the control were relatively modest, with a high degree of similarity.

### 3.3. Effects of Different Dietary Additives on Intestinal Digestive Enzyme

**Figure 4:**
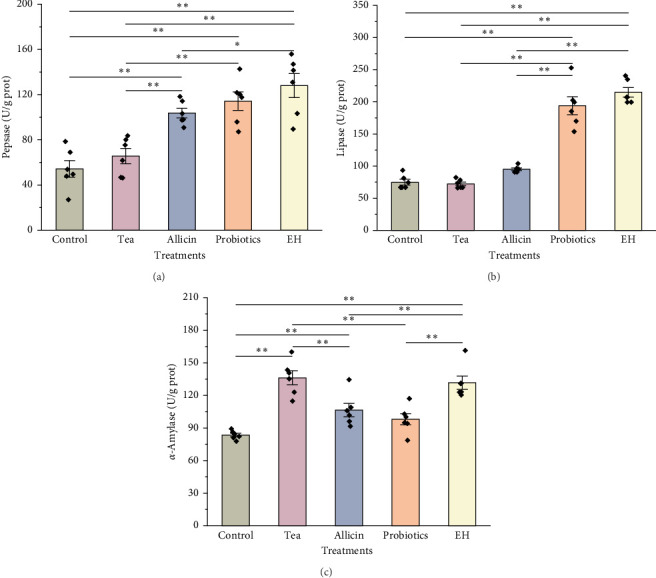
Digestive enzyme activity in intestines of juvenile sea cucumbers *Stichopus monotuberculatus* under different dietary additive treatments. (a) Pepsin, (b) lipase, and (c) *α*-amylase. Asterisk “*⁣*^*∗*^” represent a significant difference (*p*  < 0.05) and “*⁣*^*∗∗*^” represented *p*  < 0.01 between two treatments. ◆ represent the means for each replicate under one treatment.

The results of digestive physiology are illustrated in Figure 4. The pepsin activity levels in the Allicin, Probiotics, and EH groups were markedly elevated compared to those in the Control and Tea groups (*p*  < 0.01). In terms of lipase activity, the Probiotics and EH groups exhibited markedly higher values than the Control group (*p*  < 0.01), while the Tea and Allicin groups did not demonstrate a notable impact on lipase activity (*p*  > 0.05). Furthermore, the amylase activity levels in the Tea, Allicin, and EH groups surpassed those in the Control group significantly (*p*  < 0.01). In contrast, the probiotics additives did not show a significant effect on amylase activity.

### 3.4. Effects of Different Additive Inclusion on Immune-Related Enzymes Activity

**Figure 5:**
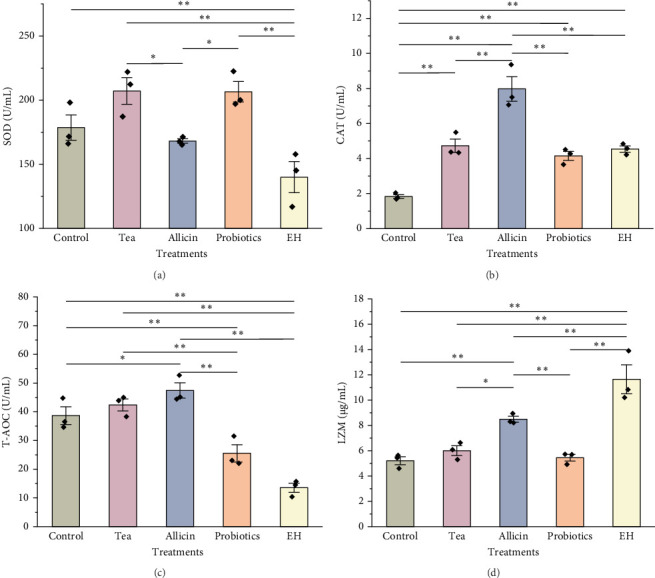
Immune enzyme activity in celomic fluid of juvenile sea cucumbers *Stichopus monotuberculatus* under different dietary additive treatments. (a) Superoxide dismutase (SOD), (b) catalase (CAT), (c) total antioxidant capacity (T-AOC), and (d) lysozyme (LZM). Asterisk “*⁣*^*∗*^” represent a significant difference (*p*  < 0.05) and “*⁣*^*∗∗*^” represented *p*  < 0.01 between two treatments. ◆ represent the means for each replicate under one treatment.

Immune-related enzyme activities in the celomic fluid of juvenile sea cucumbers are presented in Figure 5. The level of SOD was slightly elevated in the groups supplemented with partridge tea powder and probiotics compared to the Control group. Whereas it was significantly lower in the EH group (140.00 U/mL) than in the Control group (178.60 U/mL; *p*  < 0.01). For CAT activity in the celomic fluid, all four feed additives inclusion showed significantly higher levels than the Control group (*p*  < 0.01). The Allicin group exhibited a significantly higher level in antioxidant capacity (T-AOC) than the Control group (*p*  < 0.05), while both the Probiotics group and the EH group showed significantly lower levels than the Control group with a highly significant level (*p*  < 0.01). LZM activity was significantly higher in the Allicin group and EH group compared to the Control group (*p*  < 0.01).

## 4. Discussion

### 4.1. Enhancement of Growth Performance

This study found that plant-derived additives, specifically partridge tea powder and allicin, significantly improved growth performance in juvenile sea cucumbers compared to the microbial additives. Over the first 28 days, the GR in the Tea group was notably higher than that of both the Control group and other treatments. This indicates that the active ingredients in partridge tea powder may promote growth in juvenile sea cucumbers. Previous studies have shown that partridge tea is rich in phenols, alkaloids, ketones, terpenes, and other active components known for their antioxidant properties, growth promotion potential, and muscle quality enhancement. These factors could contribute to the increased GR observed in sea cucumbers [33–35]. Hu et al. [32] demonstrated that supplementing feed with 300 mg/kg of tea polyphenol enhanced growth and feed utilization of juvenile bream (*Megalobrama amblycephala*), while also improving the antioxidant capacity of the liver and intestinal tract. However, the GR of the Tea group declined during the later stages, specifically from 28 to 56 days. This decline may be due to the accumulation of alkaloids and other substances in the culture water, which could partially inhibit the growth of juvenile sea cucumbers. These findings highlight the need to assess the suitability and potential side effects of partridge tea powder at different growth stages when used as a feed additive.

The Allicin group exhibited a consistent rate of weight gain throughout the experimental period, suggesting a sustained positive impact of allicin on the growth of *S. monotuberculatus*. Allicin possesses antibacterial and antiviral properties that enhance the nonspecific immunity of aquatic animals [36, 37]. Consequently, the use of allicin contributes to the health of sea cucumbers, facilitating their growth. Li et al. [31] demonstrated that adding 200 mg/kg of allicin significantly boosted the GR of fish larvae. Other research indicated that the addition of 40 mg/kg of allicin markedly enhanced protein utilization, SGR, and FCR in sturgeon [38]. Huang et al. [39] demonstrated that the relative GR of sea cucumbers *A. japonicas* was significantly higher in groups fed allicin compared to the Control group. This suggests that dietary allicin can stimulate the growth of sea cucumbers, aligning with the results of this study. Consequently, the supplementation of allicin to the feed of juvenile *S. monotuberculatus* can reduce feed costs while improving culture efficiency.

The probiotics used in this study are based on EMs, which include yeast, lactic acid bacteria, and photosynthetic bacteria. These probiotics are known for their ability to repel pathogenic microorganisms, enhance host immunity, and improve intestinal health, making them widely adopted in aquaculture due to their safety and effectiveness [40–42]. During the experimental period, the Probiotics group exhibited a slightly higher GR than the Control group, potentially attributed to the active ingredients in the probiotics. Additionally, probiotics significantly enhance water quality, optimizing the growth environment for sea cucumbers and promoting juvenile growth [43–45]. In a study on *A. japonicus*, Li et al. [46] found that two strains of lactic acid bacteria sourced from marine fish notably enhanced sea cucumber growth. Wang and Xu [47] also studied the effect of complex probiotics on common carp (*Cyprinus carpio*), reporting that probiotics significantly improved growth performance and digestive enzyme activity. The superior growth performance and lower FCR observed in the Probiotics group suggest that incorporating probiotics as a feed additive in commercial aquaculture can enhance efficiency and reduce feed costs.

EH is a natural bioactive substance rich in amino acids, vitamins, minerals, and various enzymes, which have been shown to promote growth, improve body composition, and enhance digestion in aquatic organisms [27]. Although this study found no significant effect on growth performance, further analyses revealed its potential positive contribution to nutrient accumulation.

A decrease in IR may indicate that feeds containing allicin and probiotics increased satiety and reduced the feeding frequency of sea cucumbers. Allicin is known in aquaculture for enhancing feed palatability, which stimulates appetite and ultimately increases feed intake for its strong flavor [48]. Previous studies have demonstrated that moderate amount of allicin can significantly enhance the feeding behavior of *Litopenaeus vannamei* and *Oreochromis niloticus* [49]. Regarding FCR and SGR, the Tea group, Probiotics group, and Allicin group all showed improved feed utilization and GRs compared to the Control group.

### 4.2. Improvement of Nutritional Composition in Tissue

The results revealed that the fatty acid content in the Allicin group was lower compared to that in the Control group. Allicin is recognized for its role in regulating lipid metabolism, promoting fatty acid oxidation and energy expenditure, which ultimately leads to reduced body fat accumulation [50]. It is presumed that allicin influences lipid metabolism, which may alter the composition of SFAs and the energy storage structure in sea cucumbers. The results indicated a significant reduction in the crude fat content in Allicin group compared to other treatment groups, highlighting a notable role of allicin in modulating lipid metabolism and enhancing muscle mass. Previous studies demonstrated a remarkable increase in muscle mass in various aquaculture species due to allicin, consistent with the findings of this study [49].

Amino acid analysis revealed that the ratio of EAAs to total amino acids (TAAs) in the body wall tissues of sea cucumbers was greater in the Allicin group compared to other treatments. This suggests that allicin may facilitate the accumulation of EAAs, which are crucial for the growth and development of sea cucumbers. The provision of EAAs is crucial for the health and growth of aquatic organisms, particularly juveniles, who have a high demand for these nutrients [51, 52]. The Allicin group exhibited a decrease in fatty acid content and an increase in amino acid content, suggesting that allicin may positively affects protein metabolism, and thereby, enhances protein synthesis efficiency or facilitates amino acid uptake and utilization in *S. monotuberculatus*. Studies have shown that supplementing diets with specific EAAs, such as branched-chain amino acids (BCAAs), significantly decrease lipid synthesis, promotes fatty acid degradation, and reduce lipid accumulation [53]. In contrast, the Tea group and Probiotics group did not significantly affect the nutrient composition of body wall tissues. This indicates differences in the mechanisms and effectiveness of these feed additives compared to allicin, a distinction supported by PCA.

Nutritional analyses revealed that sea cucumbers fed with EH were remarkably higher in crude protein and crude fat contents than other groups, implying that EH may have a positive effect on the nutrient accumulation of sea cucumbers. Previous studies have indicated that probiotics incorporated into fermented feeds can elevate soluble protein and small molecule peptide levels, as well as facilitate the breakdown of dietary fiber. These essential processes may foster the synthesis and accumulation of nutrients in sea cucumbers [54, 55]. While improved nutrition typically correlates with enhanced growth, this enhanced accumulation did not directly translate into improved growth performance in the EH group. Research has shown that a moderate increase in dietary lipid levels can enhance weight gain and the SGR of fish [56]. Conversely, excessive lipids can impair fish's ability to digest and absorb fatty acids, resulting in reduced GRs [57]. Liao et al. [58] conducted a study on *A. japonicus* that identified a decreasing trend in both weight gain and SGR of sea cucumbers as dietary lipid levels increased. This decline may be attributed to the fact that the natural diet of sea cucumbers typically consists of low lipid levels, and they do not require elevated dietary lipid levels for optimal growth performance.

The results also indicated that the levels of fatty acids and EAAs in the body wall of sea cucumbers in the EH group were significantly higher, particularly the levels of BCAAs. BCAAs, including isoleucine, leucine, and valine, are EAAs that play important structural roles. BCAAs are primarily involved in physiological processes such as gluconeogenesis, lipogenesis, and both protein synthesis and catabolism [59]. Research has shown that when the levels of EAAs in fish are elevated, especially BCAAs, the GR can be significantly enhanced [60]. However, when the content of BCAAs is too high in the body, this accumulation could be due to defective BCAA catabolism caused by reduced expression of branched-chain ketoacid dehydrogenase (BCKDH) [61]. It has also been found that when animals have excess energy in their bodies, they inhibit BCKDH expression, resulting in elevated levels of both BCAAs and branched-chain *α*-keto acids (BCKAs) [62]. In the previous discussion, it was inferred that diets supplemented with EH contained lipid levels that were too high for sea cucumbers. Therefore, it was hypothesized that sea cucumbers fed with EH experienced an overnutritional condition, leading to an increase in BCAA levels in the body.

### 4.3. Effects on Intestinal Digestive Physiology

The experimental results revealed that the partridge tea powder might not significantly enhance the digestive performance of *S. monotuberculatus*. There was no remarkable difference in protease and lipase activities in Tea group compared to the Control group. However, it has been suggested that the amino acids in terrestrial plants used as feed additives are not balanced for marine species, leading to reduced digestibility [63, 64]. In addition, it has been speculated that tea polyphenols may act as an antinutritional factor with inhibitory effects on digestive enzymes, potentially due to the synergistic effect of hydrophobic association and hydrogen bond formation between tea polyphenols and enzymes [65]. Whereas the present study did not observe any appreciable inhibitory effect on the digestive properties, it is hypothesized that the experimental addition of partridge tea powder may not have released enough components to modify the activity of digestive enzymes.

Allicin, however, dramatically increases the activity of digestive enzymes in sea cucumbers. This effect is attributed to the antimicrobial properties of allicin, which help maintain the balance of intestinal microorganisms and further promote digestive enzyme activity [66]. The primary biological effect of allicin results from its rapid reaction with thiol-containing proteins, which effectively activate various proteases. The significant improvement of pepsin activity in the allicin group compared to the control group may reflect the role of allicin in positively regulating the gastric environment of sea cucumbers. Pepsin functions optimally in acidic conditions, and allicin likely enhances pepsin activity by curbing harmful bacterial growth and reducing intragastric pH levels [67].

The research findings demonstrate that the Probiotics group significantly increased the activities of pepsin and lipase, while also exhibiting a moderate enhancement in *α*-amylase activity. A study affirmed that EMs could boost the GR by elevating protease and amylase levels in sea cucumbers [68]. This enhancement is attributed to the direct addition of a probiotic blend to the feed, enabling colonization in the intestinal tract of aquatic animals, thereby, improving the internal environment and promoting growth [69]. In a study on *A. japonicus*, Wang et al. [70] incorporated the probiotic *Rhodotorula benthica* D30 into sea cucumber bait, resulting in considerable enhancements in amylase, cellulase, and protease activities. These findings indicate the effectiveness of this probiotic for aquaculture and are consistent with the digestive physiology observed in *S. monotuberculatus* [70]. Moreover, Wang and Xu [47] supplemented various probiotic compositions into basal diets for *C. carpio*, demonstrating that blended probiotic forms were more effective in enhancing growth performance and digestive enzyme activity compared to individual forms.

The use of EH significantly enhanced the activity of digestive enzymes in the intestinal tract of the sea cucumber, resulting in improved digestive performance. Analysis of the crude composition of the feedstuffs revealed that the crude protein content in the feed supplemented with probiotics and EH was notably higher than in other components. This suggests that crude protein content positively affects the digestive performance of sea cucumbers. Research on *A. japonicus* has shown that high-protein feeds can increase the digestive load, resulting in adverse effects [71]. Similarly, it is essential that the protein content of feeds align with the ideal pattern necessary for the optimum growth of aquatic organisms. Such alignment allows the animals to effectively utilize nutrients for growth and minimizes the discharge of extra nitrogen into the water [72, 73]. This demonstrates that the high content of crude protein in the EH group and Probiotic group provided a more desirable amino acid pattern for the growth of *S. monotuberculatus*.

### 4.4. Effects on Immune-Related Enzymes in Celomic Fluid

Echinoderms primarily rely on cellular and humoral immunity to combat external threats, where nonspecific immune enzymes play a crucial role in the immune defense of sea cucumbers [74, 75]. The Tea group exhibited significant enhancements in SOD and CAT activities compared to the Control group, without inhibiting T-AOC or LZM activity, indicating improved immune performance in sea cucumbers due to partridge tea powder. The tea polyphenols present in partridge tea powder were found to have considerable antioxidant effects that could decrease ROS levels, enhance the activities of antioxidant enzymes, and reduce oxidative stress [76]. Among these, epigallocatechin 3-gallate (EGCG) and quercetin can scavenge free radicals and reactive oxygen species, effectively increasing the antioxidant capacity of the organism [77, 78]. Research on the optimal incorporation of tea polyphenols in high-fat diets for juvenile turbot fish revealed that a 0.02% level promoted growth and enhanced serum antioxidant capacity [79]. This finding aligns with the results of this study, emphasizing the diverse potential applications of tea polyphenols in bolstering antioxidant defenses in aquatic animals.

The CAT activity, T-AOC, and LZM activity in sea cucumbers from the Allicin group were markedly higher than those in the Control group. This observation suggests that allicin likely plays a pivotal role in augmenting the antioxidant capacity and nonspecific immunity of sea cucumbers, thereby, enhancing their resilience to environmental stressors. The beneficial impact of allicin is possibly attributed to its sulfur compounds, known for their antioxidant and anti-inflammatory properties [80]. Studies on *Oreochromis mossambicus* have indicated that incorporating allicin into feed enhances the organism's antioxidant capacity, leading to a favorable physiological state and sustained metabolic activity [81]. An investigation on large yellow croaker (*Larimichthys crocea*) revealed a notable increase in CAT activity when 0.02% allicin was added to the diet. The study also proposed that integrating allicin into the feed could potentially enhance larval survival and growth by stimulating intestinal development, mitigating inflammation, and boosting appetite [82].

In contrast, the findings from the Probiotic and EH groups demonstrated that both interventions had multifaceted impacts on the immune enzyme activities of sea cucumbers. The incorporation of probiotics notably elevated the levels of SOD and CAT levels, while constraining T-AOC. On the other hand, the inclusion of EH significantly enhanced CAT and LZM levels, while also suppressing T-AOC. These results suggest that probiotics and EH can enhance the activity of specific immune enzymes to a certain extent. Gao et al. [83] found that LZM activity in various tissues did not further increase, but rather declined with continual increases in dietary protein levels in *Scophthatmus maximus*. This indicates that a high dietary protein level is unnecessary for enhancing nonspecific immune function in fish [83]. In a study on *A. japonicus*, they had a better phagocytic ability and foreign objects removal when dietary protein levels were low, compared to those fed on a high-protein diet [84]. Studies on *A. japonicus* have revealed that an excessive protein intake from animal sources in the diet can heighten the metabolic load of protein on the organism, resulting in the inhibition of immune functions [85]. It can be inferred that the dietary protein content required to improve the nonspecific immunity of sea cucumbers similarly does not need to be high. Therefore, although the high level of crude protein contained in probiotics and EH benefits digestive enzyme activity, the protein content of these additives does not seem to have a completely positive effect on immune enzyme activity.

In the aquaculture industry, the combined use of multiple feed additives is expected to produce stronger biological effects than the use of a single additive. Further investigation is needed to determine whether the combined application of the additives used in this study will synergistically enhance their individual benefits to achieve the desired effect. Additionally, feed additives that remain in culture waters can be released into the surrounding aquatic ecosystems, potentially disrupting their natural structure and function. For instance, the use of probiotics may alter the natural bacterial composition of the environment. Therefore, the potential ecological impacts of these additives, especially with long-term application, warrant further investigation.

## 5. Conclusion

This study evaluated the effects of four feed additives, partridge tea powder, allicin, probiotics, and EH, on the nutrient composition, growth performance, digestion, immunity, and antioxidant capacity of juvenile *S. monotuberculatus*. The results provided valuable insights into addressing slow growth and high mortality rates in juvenile sea cucumber aquaculture. Supplementation with plant-derived additives partridge tea powder and allicin significantly enhanced growth and immunity. In contrast, microbial additives, probiotics, and EH improved nutrient composition and digestibility, but had limited effects on growth performance and immunity. Moreover, using these readily available and inexpensive plant-derived and microbial additives could reduce the costs of sea cucumber farming. These findings pave the way for developing innovative feed additives aimed at optimizing farming practices and improving the efficiency and sustainability of tropical juvenile sea cucumber cultivation.

## Figures and Tables

**Table 1:** Ingredients and proximate biochemical composition of the basal feed.

Constituent	Composition
Ingredients (%)	
* Sargassum denticarpum* meal	25
* Sargassum thunbergii* meal	25
Formula feed	25
Sea mud	25
Proximate composition (%)	
Crude protein	13.69
Crude fat	1.92
Ash	7.31

**Table 2:** The composition of experimental dietary additives.

Dietary additives	Crude protein (% DW)	Lipid (% DW)	Ash (% DW)	Moisture (%)
Partridge tea powder (Tea)	11.29	3.23	32.60	0.05
Allicin	2.29	0	91.40	0.03
Probiotics	61.68	1.28	5.83	84.27
EH	37.72	9.67	28.38	95.93

*Note:* Crude protein, crude lipid, and ash content were expressed as ratios on a dry weight (DW) basis.

Abbreviations: DW, dry weight; EH, earthworm hydrolysate.

**Table 3:** Composition of feed additives under different feeding groups.

Group	Diet composition
Control	Basal feed
Tea	Basal feed + partridge tea powder (500 mg/kg)
Allicin	Basal feed + allicin (100 mg/kg)
Probiotics	Basal feed + probiotics (20 mL/kg)
EH	Basal feed + earthworm hydrolysate (100 mL/kg)

**Table 4:** Growth performance of juvenile sea cucumber *Stichopus monotuberculatus*.

Growth performance	Control	Tea	Allicin	Probiotics	EH
Initial body weight (*W*_0_)/g	2.17 ± 0.05	2.09 ± 0.08	2.44 ± 0.11	2.48 ± 0.16	2.47 ± 0.17
Final body weight (*W*_t_)/g	4.27 ± 0.07^a,b^	4.56 ± 0.07^a^	4.66 ± 0.40^a^	4.53 ± 0.40^a^	3.52 ± 0.10^b^
14-day GR (%)	14.91 ± 0.68^b^	43.15 ± 0.90^a^	17.94 ± 1.77^b^	17.4 ± 1.90^b^	13.10 ± 1.64^b^
28-day GR (%)	15.35 ± 0.35^b^	22.79 ± 4.01^a^	19.98 ± 1.87^a,b^	18.24 ± 1.96^a,b^	7.35 ± 0.22^c^
42-day GR (%)	19.51 ± 0.44^b^	13.63 ± 2.64^c^	22.29 ± 0.55^a,b^	26.24 ± 2.80^a^	10.13 ± 1.09^c^
56-day GR (%)	18.70 ± 0.10^a^	11.90 ± 1.63^b^	20.33 ± 2.21^a^	16.95 ± 1.31^a^	8.93 ± 1.56^b^
GR (%)	88.05 ± 1.42^c^	123.14 ± 2.92^a^	108.33 ± 7.13^a,b^	105.09 ± 8.44^b,c^	45.70 ± 4.01^d^
SR (%)	90.00	96.67 ± 3.33	90.00 ± 10	83.33 ± 3.33	93.33 ± 6.67

*Note:* Data were presented as mean ± SEM, *n* = 3. Treatments marked with different letters are significantly different (*p*  < 0.05).

Abbreviations: EH, earthworm hydrolysate; GR, growth rate; SEM, standard error; SR, survival rate.

**Table 5:** Nutritional composition of *Stichopus monotuberculatus* under different treatments.

Treatments	Control (% DW)	Tea (% DW)	Allicin (% DW)	Probiotics (% DW)	EH (% DW)
Crude protein	51.24 ± 0.31^b^	50.98 ± 0.63^b^	51.53 ± 0.35^b^	51.09 ± 0.46^b^	54.08 ± 0.28^a^
Crude lipid	2.14 ± 0.03^b^	2.12 ± 0.07^b^	1.92 ± 0.08^c^	2.26 ± 0.03^b^	2.47 ± 0.06^a^
Ash	30.90 ± 0.03	31.30 ± 0.16	31.18 ± 0.16	31.33 ± 0.16	30.78 ± 0.29
Moisture	90.68 ± 0.48	90.70 ± 0.47	90.94 ± 0.23	90.53 ± 0.47	90.34 ± 0.07

*Note:* Data were presented as mean ± SEM, *n* = 3. Treatments marked with different letters are significantly different (*p* < 0.05).

Abbreviations: DW, dry weight; EH, earthworm hydrolysate; SEM, standard error.

**Table 6:** Amino acid profile in the tissue of juvenile sea cucumber under different dietary additives treatments.

Amino acid (g/kg)	Control	Tea	Allicin	Probiotics	EH
Asp	6.15 ± 0.51^a,b^	6.04 ± 0.26^a,b^	5.86 ± 0.23^b^	6.51 ± 0.44^a,b^	7.12 ± 0.32^a^
Glu	9.81 ± 0.85^a,b^	9.61 ± 0.43^b^	9.90 ± 0.35^a,b^	10.79 ± 0.70^a,b^	11.68 ± 0.54^a^
Ser	2.64 ± 0.24	2.61 ± 0.12	2.68 ± 0.09	2.91 ± 0.19	3.17 ± 0.16
Gly	7.22 ± 0.69	7.11 ± 0.35	7.32 ± 0.07	7.98 ± 0.49	7.88 ± 0.38
His	1.52 ± 0.06^b^	1.49 ± 0.02^b^	1.63 ± 0.03^a,b^	1.62 ± 0.04^a,b^	1.74 ± 0.06^a^
Arg*⁣*^*∗*^	4.69 ± 0.38	4.61 ± 0.18	4.72 ± 0.15	5.07 ± 0.30	5.32 ± 0.23
Thr*⁣*^*∗*^	3.28 ± 0.26	3.29 ± 0.20	3.30 ± 0.14	3.61 ± 0.22	3.89 ± 0.16
Ala	4.23 ± 0.36	4.16 ± 0.19	4.21 ± 0.09	4.63 ± 0.29	4.74 ± 0.22
Pro	4.22 ± 0.40	4.18 ± 0.21	4.38 ± 0.04	4.68 ± 0.28	4.82 ± 0.22
Tyr*⁣*^*∗*^	1.53 ± 0.13	1.54 ± 0.07	1.68 ± 0.07	1.64 ± 0.06	1.80 ± 0.07
Val*⁣*^*∗*^	2.62 ± 0.21^b^	2.57 ± 0.09^b^	2.76 ± 0.03^a,b^	2.83 ± 0.12^a,b^	3.13 ± 0.19^a^
Met*⁣*^*∗*^	1.20 ± 0.08	1.17 ± 0.06	1.20 ± 0.09	1.23 ± 0.05	1.27 ± 0.02
Ile*⁣*^*∗*^	2.14 ± 0.15^b^	2.10 ± 0.05^b^	2.26 ± 0.01^a,b^	2.35 ± 0.09^a,b^	2.57 ± 0.16^a^
Leu*⁣*^*∗*^	3.11 ± 0.22^b^	3.07 ± 0.09^b^	3.37 ± 0.03^a,b^	3.47 ± 0.15^a,b^	3.83 ± 0.25^a^
Phe*⁣*^*∗*^	1.89 ± 0.14^b^	1.89 ± 0.05^b^	2.08 ± 0.01^a,b^	2.14 ± 0.08^a,b^	2.41 ± 0.15^a^
Lys*⁣*^*∗*^	2.64 ± 0.16^b^	2.55 ± 0.05^b^	2.83 ± 0.06^a,b^	2.88 ± 0.10^a,b^	3.21 ± 0.22^a^
EAA	24.87 ± 1.81^b^	24.45 ± 0.77^b^	26.28 ± 0.38^a,b^	27.20 ± 1.21^a,b^	29.47 ± 1.58^a^
TAA	58.90 ± 4.78	58.00 ± 2.32	60.19 ± 1.27	64.33 ± 3.58	68.59 ± 3.23
EAA/TAA (%)	42.28 ± 0.54^b^	42.19 ± 0.36^b^	43.68 ± 0.30^a^	42.34 ± 0.45^a,b^	42.94 ± 0.36^a,b^

*Note:* Data were presented as mean ± SEM, *n* = 3. Treatments marked with different letters are significantly different (*p*  < 0.05). The asterisk (*⁣*^*∗*^) in the first column indicates the essential amino acids.

Abbreviations: Ala, alanine; Arg, arginine; Asp, aspartic acid/asparagine; EAA, essential amino acid; EH, earthworm hydrolysate; Glu, glutamic acid/glutamine; Gly, glycine; His, histidine; Ile, isoleucine; Leu, leucine; Lys, lysine; Met, methionine; Phe, phenylalanine; Pro, proline; SEM, standard error; Ser, serine; TAA, total amino acid; Thr, threonine; Tyr, tyrosine; Val, valine.

**Table 7:** Fatty acid profile in the tissue of juvenile sea cucumber under different dietary additives treatments.

Fatty acid (mg/kg)	Control	Tea	Allicin	Probiotics	EH
C14:0	9.84 ± 0.20^a,b^	10.65 ± 0.56^a^	9.37 ± 0.20^b^	9.76 ± 0.26^a,b^	10.03 ± 0.02^a,b^
C16:0	92.31 ± 2.79^a,b^	93.33 ± 4.54^a,b^	87.39 ± 3.59^b^	99.41 ± 3.09^a^	101.57 ± 0.17^a^
C18:0	84.53 ± 2.79^a,b,c^	82.70 ± 4.15^b,c^	78.58 ± 5.68^c^	91.87 ± 1.86^a,b^	94.82 ± 0.39^a^
C20:0	11.14 ± 0.47^a^	10.57 ± 0.10^a,b^	8.68 ± 1.32^b^	10.01 ± 0.15^a,b^	11.18 ± 0.19^a^
C21:0	11.65 ± 0.61^a,b^	10.57 ± 0.17^a,b^	8.46 ± 1.95^b^	11.53 ± 0.59^a,b^	12.53 ± 0.36^a^
C22:0	9.21 ± 0.09^b,c^	10.26 ± 0.34^a,b^	8.90 ± 0.72^c^	9.01 ± 0.14^b,c^	10.66 ± 0.37^a^
C14:1*n*−5	9.30 ± 0.45	9.40 ± 0.72	8.26 ± 0.60	8.97 ± 0.13	8.76 ± 0.04
C16:1*n*−9c	8.17 ± 0.56	7.72 ± 0.14	7.53 ± 0.39	7.83 ± 0.12	7.98 ± 0.03
C18:1*n*−9c	9.48 ± 0.98	9.31 ± 0.45	6.24 ± 2.36	9.12 ± 0.59	10.02 ± 0.74
C18:1*n*−9t	12.80 ± 0.98	12.28 ± 0.55	11.79 ± 1.10	12.64 ± 0.66	12.90 ± 0.39
C20:1	28.67 ± 0.55	28.46 ± 1.17	28.02 ± 3.00	26.43 ± 1.43	29.86 ± 0.49
C24:1*n*−9	11.20 ± 0.78	10.55 ± 0.59	9.21 ± 1.16	10.46 ± 0.49	11.64 ± 0.45
C18:2*n*−6c	19.66 ± 3.34	16.39 ± 1.06	17.94 ± 1.42	19.27 ± 1.14	20.83 ± 1.84
C20:2	19.92 ± 0.91^a^	18.84 ± 0.06^a,b^	14.93 ± 2.83^b^	18.28 ± 0.44^a,b^	20.93 ± 0.47^a^
C20:4*n*−6	58.61 ± 1.35^a,b^	58.78 ± 2.29^a,b^	41.48 ± 15.10^b^	58.87 ± 4.39^a,b^	68.52 ± 2.77^a^
C20:5*n*−3	69.36 ± 2.42^a,b^	67.81 ± 1.48^a,b^	50.25 ± 17.39^b^	73.31 ± 6.09^a,b^	82.15 ± 4.23^a^
SFA	218.68 ± 6.64^a,b^	218.07 ± 8.82^a,b^	201.38 ± 10.54^b^	231.59 ± 4.60^a^	240.79 ± 0.33^a^
UFA	247.17 ± 12.32^a,b^	239.54 ± 4.87^a,b^	195.65 ± 37.78^b^	245.18 ± 11.33^a,b^	273.60 ± 10.76^a^

*Note:* Data were presented as mean ± SEM, *n* = 3. Treatments marked with different letters are significantly different (*p*  < 0.05).

Abbreviations: EH, earthworm hydrolysate; SEM, standard error; SFA, saturated fatty acid; UFA, unsaturated fatty acid.

## Data Availability

The data are available on request from the authors.

## References

[1] Yu Z., Hu C., Zhou Y., Li H., Peng P. (2012). Survival and Growth of the Sea Cucumber, *Holothuria leucospilota*, Brandt: A Comparison Between Suspended and Bottom Cultures in a Subtropical Fish Farm During Summer. *Aquaculture Research*.

[2] Vidal-Ramirez F., Dove S. (2016). Diurnal Effects of *Holothuria atra* on Seawater Carbonate Chemistry in a Sedimentary Environment. *Journal of Experimental Marine Biology and Ecology*.

[3] Ma B., Liu Y., Pan W. (2022). Integrative Application of Transcriptomics and Metabolomics Provides Insights Into Unsynchronized Growth in Sea Cucumber (*Stichopus monotuberculatus*). *International Journal of Molecular Sciences*.

[4] Jia J., Chen J. (2001). *Sea Farming and Sea Ranching in China*.

[5] Liao Y. (1997). *Fauna Sinica: Phylum Echinodermata Class Holothuroidea*.

[6] Yuan L., Hu C., Zhang L., Xia J. (2013). Population Genetics of a Tropical Sea Cucumber Species (*Stichopus monotuberculatus*) in China. *Conservation Genetics*.

[7] Lane D. J. W., Limbong D. (2015). Catastrophic Depletion of Reef-Associated Sea Cucumbers: Resource Management/Reef Resilience Issues for an Indonesian Marine Park and the Wider Indo-Pacific. *Aquatic Conservation: Marine and Freshwater Ecosystems*.

[8] Han Q., Keesing J. K., Liu D. (2016). A Review of Sea Cucumber Aquaculture, Ranching, and Stock Enhancement in China. *Reviews in Fisheries Science & Aquaculture*.

[9] Cheng C., Wu F., Ren C. (2021). Aquaculture of the Tropical Sea Cucumber, *Stichopus monotuberculatus*: Induced Spawning, Detailed Records of Gonadal and Embryonic Development, and Improvements in Larval Breeding by Digestive Enzyme Supply in Diet. *Aquaculture*.

[10] Chan Q., Wang F., Han Y. (2022). An Investigation on Dietary Chromium Picolinate Supplementation in the Juvenile Sea Cucumber *Apostichopus japonicus*: Growth, Digestive Enzyme Activity, Growth-Related Genes Expression, Immune and Antioxidant Capacity. *Aquaculture Reports*.

[11] Purcell S. W., Hair C. A., Mills D. J. (2012). Sea Cucumber Culture, Farming and Sea Ranching in the Tropics: Progress, Problems and Opportunities. *Aquaculture*.

[12] Williams A. J., Allain V., Nicol S. J. (2015). Vertical Behavior and Diet of Albacore Tuna (*Thunnus alalunga*) Vary With Latitude in the South Pacific Ocean. *Deep Sea Research Part II: Topical Studies in Oceanography*.

[13] Santamaría-Miranda A., Dumas S., Pérez-Urbiola J. C. (2021). Fatty Acid Composition and Spawning Quality in Wild and Captive Broodstock of Pacific Red Snapper *Lutjanus peru*. *Aquaculture*.

[14] Lawrence J. M. (2013). *Sea Urchins: Biology and Ecology*.

[15] Seo J. Y., Shin I. S., Lee S. M. (2011). Effect of Dietary Inclusion of Various Plant Ingredients as an Alternative for *Sargassum thunbergii* on Growth and Body Composition of Juvenile Sea Cucumber *Apostichopus japonicu*s. *Aquaculture Nutrition*.

[16] Yu Y., Ding P., Qiao Y. (2023). The Feces of Sea Urchins as Food Improves Survival, Growth, and Resistance of Small Sea Cucumbers *Apostichopus japonicus* in Summer. *Scientific Reports*.

[17] Ma Y., Sun F., Zhang C., Bao P., Cao S., Zhang M. (2014). Effects of Pseudoalteromonas sp. BC228 on Digestive Enzyme Activity and Immune Response of Juvenile Sea Cucumber (*Apostichopus japonicus*). *Journal of Ocean University of China*.

[18] Lu T., Wang C., Guo M., Li C., Shao Y. (2024). Effects of Dietary Vibrio sp. 33 on Growth, Innate Immunity, Gut Microbiota Profile and Disease Resistance Against *Vibrio splendidus* of Juvenile Sea Cucumber *Apostichopus japonicu*s. *Developmental Comparative Immunology*.

[19] Yan W., Li J., Zheng D., Friedman C., Wang H. (2019). Analysis of Genetic Population Structure and Diversity in *Mallotus oblongifolius* Using ISSR and SRAP Markers. *PeerJ*.

[20] Fu J., Yu H. D., Wu L., Zhang C., Yun Y. H., Zhang W. (2021). Authentication of Geographical Origin in Hainan Partridge Tea (*Mallotus obongifolius*) by Stable Isotope and Targeted Metabolomics Combined with Chemometrics. *Foods*.

[21] Zeb A. (2020). Concept, Mechanism, and Applications of Phenolic Antioxidants in Foods. *Journal of Food Biochemistry*.

[22] Guo H., Lin W., Wang L. (2020). The Supplementation of Dietary Selenium Yeast and Green Tea-Derived Polyphenols Improves Antioxidant Capacity and Immune Response in Juvenile Wuchang Bream Under Ammonia Stress. *Aquaculture Research*.

[23] Wang X., Qian Y., Gu T. (2021). Dietary Tea Polyphenols Change Flesh Quality With Dose-Related Manner in the GIFT Tilapia Fed With a High-Fat Diet. *Aquaculture Nutrition*.

[24] Ma Y., Zhou X., Jiang W. (2024). Tea Polyphenols Protect Against *Flavobacterium columnare*-Induced Gill Injury via Suppression of Oxidative Stress, Inflammation, and Apoptosis in Grass Carp. *International Journal of Biological Macromolecules*.

[25] Rocío V.-G., Asunción L.-L., Francisco V.-A., Francesco C., Marcel M.-P. (2021). Exploring the Garlic (*Allium sativum*) Properties for Fish Aquaculture. *Fish Physiology and Biochemistry*.

[26] Nugraha T. A., Isnansetyo A., Triyanto, Djalil M. (2022). Fermented Earthworms as a Feed Additive Enhances Non-Specific Immune Response in Catfish (*Clarias gariepinus*). *Aquaculture International*.

[27] Zhou J., Ma Z., Li Y., Naixia W., Wu H. (2022). Effect of Fermented Earthworm Broth on Production Performance, Egg Quality and Lipid Index of Kangle Yellow Chicken. *Chinese Journal of Veterinary Medicine*.

[28] Wang Y.-B., Li J.-R., Lin J. (2008). Probiotics in Aquaculture: Challenges and Outlook. *Aquaculture*.

[29] Liu Y., Bai Q., Wang L., Li C., Li Y., Liu B. (2023). Effects of Dietary Bacillus Baekryungensis on Body Wall Nutrients, Digestion and Immunity of the Sea Cucumber *Apostichopus japonicus*. *Fisheries Science*.

[30] Wang X., Li S., Dong Y. (2023). Effects of Dietary *Bacillus licheniformis* and Combined Herbs Extracts Supplementation on Physiological and Immune Characteristics, Microbial Community, and Vibriosis Resistance of *Apostichopus japonicus*. *Aquaculture Research*.

[31] Li H., Huang B., Liu B., Liu B., Wang W. (2020). Effects of Allicin on Growth and Disease Resistance in Turbot. *Fisheries Science*.

[32] Hu S., Mu Q., Lin Y., Miao L., Liu B., Dong Z. (2023). Effects of Different Supplementation of Dietary Tea Polyphenols on Growth Performance, Feed Utilization and Antioxidant Capacity of *Megalobrama amblycephala*. *Journal of Fisheries of China*.

[33] Hwang J.-H., Lee S.-W., Rha S.-J. (2013). Dietary Green Tea Extract Improves Growth Performance, Body Composition, and Stress Recovery in the Juvenile Black Rockfish, *Sebastes schlegeli*. *Aquaculture International*.

[34] Ji R., Li Y., Li X. (2018). Effects of Dietary Tea Polyphenols on Growth, Biochemical and Antioxidant Responses, Fatty Acid Composition and Expression of Lipid Metabolism Related Genes of Large Yellow Croaker (*Larimichthys crocea*). *Aquaculture Research*.

[35] Liu H., Guan H., He F. (2023). Therapeutic Actions of Tea Phenolic Compounds Against Oxidative Stress and Inflammation as Central Mediators in the Development and Progression of Health Problems: A Review Focusing on microRNA Regulation. *Critical Reviews in Food Science and Nutrition*.

[36] Fall J., Tanekhy M. (2015). The Effect of Allicin on Innate Immune Genes of Common Carp (*Cyprinus carpio* L). *Journal of Applied Biotechnology*.

[37] Fall J., Tanekhy M. (2015). The Effect of Allicin on Innate Immune Genes of Common Carp (*Cyprinus carpio* L). *Journal of Applied Biotechnology*.

[38] Wu C. (2021). Effects of Allicin on Growth Performance and Body Composition of Sturgeon. *China Feed*.

[39] Huang X., Zhao Z., Yang Y. (2024). Dietary Allicin Improves Behavior, Physiology, Growth, and Disease Resistance in the Sea Cucumber *Apostichopus japonicus*. *Aquaculture*.

[40] Standen B., Rawling M., Davies S. (2013). Probiotic *Pediococcus acidilactici* Modulates Both Localised Intestinal-and Peripheral-Immunity in Tilapia (*Oreochromis niloticus*). *Fish & Shellfish Immunology*.

[41] Addo S., Carrias A. A., Williams M. A., Liles M. R., Terhune J. S., Davis D. A. J. A. R. (2017). Effects of *Bacillus subtilis* Strains and the Prebiotic Previda® on Growth, Immune Parameters and Susceptibility to *Aeromonas hydrophila* Infection in Nile Tilapia, *Oreochromis niloticus*. *Aquaculture Research*.

[42] Dong Y., Yang Y., Liu J. (2018). Inhibition of *Aeromonas hydrophila*-Induced Intestinal Inflammation and Mucosal Barrier Function Damage in Crucian Carp by Oral Administration of *Lactococcus lactis*. *Fish & Shellfish Immunology*.

[43] Gobi N., Vaseeharan B., Chen J. (2018). Dietary Supplementation of Probiotic *Bacillus licheniformis* Dahb1 Improves Growth Performance, Mucus and Serum Immune Parameters, Antioxidant Enzyme Activity as Well as Resistance Against *Aeromonas hydrophila* in Tilapia *Oreochromis mossambicus*. *Fish & Shellfish Immunology*.

[44] Wang A., Ran C., Wang Y. (2019). Use of Probiotics in Aquaculture of China—a Review of the Past Decade. *Fish & Shellfish Immunology*.

[45] Zhou W., Liu B., Li Y. (2021). Dietary Supplementation With, *Sporosarcina aquimarina*, MS4 Enhances Juvenile Sea Cucumber (*Apostichopus japonicus*) Growth, Immunity and Disease Resistance Against *Vibrio splendidus* Infection at Low Temperature. *Aquaculture Nutrition*.

[46] Li C., Ren Y., Jiang S. (2018). Effects of Dietary Supplementation of Four Strains of Lactic Acid Bacteria on Growth, Immune-Related Response and Genes Expression of the Juvenile Sea Cucumber, *Apostichopus japonicus*, Selenka. *Fish & Shellfish Immunology*.

[47] Wang Y., Xu Z. (2006). Effect of Probiotics for Common Carp (*Cyprinus carpio*) Based on Growth Performance and Digestive Enzyme Activities. *Animal Feed Science and Technology*.

[48] Chen J., Wang F., Yin Y., Ma X. (2021). The Nutritional Applications of Garlic (*Allium sativum*) as Natural Feed Additives in Animals. *PeerJ*.

[49] Yue Y., Chen M., Bao X. (2022). Effects of Three Feed Attractants on the Growth Performance and Meat Quality of the Largemouth Bass (*Micropterus salmoides*). *Frontiers in Marine Science*.

[50] Mahmoud R., Aziza A., Marghani B., Eltaysh R. (2019). Influence of Ginger and Garlic Supplementation on Growth Performance, Whole Body Composition and Oxidative Stress in the Muscles of Nile Tilapia (*O. Niloticus*). *Advances in Animal and Veterinary Sciences*.

[51] Qi C., Wang X., Han F. (2019). Arginine Supplementation Improves Growth, Antioxidant Capacity, Immunity and Disease Resistance of Juvenile Chinese Mitten Crab, *Eriocheir sinensis*. *Fish & Shellfish Immunology*.

[52] Moniruzzaman M., Damusaru J. H., Won S., Cho S. J., Chang K. H., Bai S. C. (2020). Effects of Partial Replacement of Dietary Fish Meal by Bioprocessed Plant Protein Concentrates on Growth Performance, Hematology, Nutrient Digestibility and Digestive Enzyme Activities in Juvenile Pacific White Shrimp, *Litopenaeus vannamei*. *Journal of the Science of Food and Agriculture*.

[53] Chen Q., Wang C., Sun Y. (2024). An Integrated Analysis of Transcriptome and Metabolome Reveals Three BCAAs Relieve Lipid Accumulation by Inhibiting Lipid Synthesis and Promoting Lipid Oxidation in the Liver of Largemouth Bass (*Micropterus salmoides*). *Aquaculture*.

[54] Van Vo B., Bui D. P., Nguyen H. Q., Fotedar R. J. A. (2015). Optimized Fermented Lupin (*Lupinus angustifolius*) Inclusion in Juvenile Barramundi (*Lates calcarifer*) Diets. *Aquaculture*.

[55] Siddik M. A. B., Julien B. B., Islam S. M. M., Francis D. S. (2024). Fermentation in Aquafeed Processing: Achieving Sustainability in Feeds for Global Aquaculture Production. *Reviews in Aquaculture*.

[56] Ding L., Zhang L., Wang J. (2009). *Effect of Dietary Lipid Level on the Growth Performance, Feed Utilization, Body Composition and Blood Chemistry of Juvenile Starry Flounder (Platichthys stellatus)*.

[57] Mohanta K. N., Subramanian S., Korikanthimath V. S. (2013). Effect of Dietary Protein and Lipid Levels on Growth, Nutrient Utilization and Whole-Body Composition of Blue Gourami, Trichogaster Trichopterus Fingerlings. *Journal of Animal Physiology and Animal Nutrition*.

[58] Liao M.-L., Ren T.-J., Chen W. (2017). Effects of Dietary Lipid Level on Growth Performance, Body Composition and Digestive Enzymes Activity of Juvenile Sea Cucumber, *Apostichopus japonicus*. *Aquaculture Research*.

[59] Ahmad I., Ahmed I., Fatma S., Peres H. (2021). Role of Branched-Chain Amino Acids on Growth, Physiology and Metabolism of Different Fish Species: A Review. *Aquaculture Nutrition*.

[60] Egerton S., Wan A., Murphy K. (2020). Replacing Fishmeal With Plant Protein in Atlantic Salmon (*Salmo salar*) Diets by Supplementation With Fish Protein Hydrolysate. *Scientific Reports*.

[61] Herman M. A., She P., Peroni O. D., Lynch C. J., Kahn B. B. (2010). Adipose Tissue Branched Chain Amino Acid (BCAA) Metabolism Modulates Circulating BCAA Levels. *Journal of Biological Chemistry*.

[62] Gannon N. P., Schnuck J. K., Vaughan R. A. (2018). BCAA Metabolism and Insulin Sensitivity—Dysregulated by Metabolic Status?. *Molecular Nutrition & Food Research*.

[63] Wilson R. P., Poe W. E., Robinson E. H. (1980). Leucine, Isoleucine, Valine and Histidine Requirements of Fingerling Channel Catfish. *The Journal of Nutrition*.

[64] Viola S., Mokady S., Arieli Y. (1983). Effects of Soybean Processing Methods on the Growth of Carp (*Cyprinus carpio*). *Aquaculture*.

[65] Dreosti I. E. (2000). Antioxidant Polyphenols in Tea, Cocoa, and Wine. *Nutrition*.

[66] Wills E. D. (1956). Enzyme Inhibition by Allicin, the Active Principle of Garlic. *Biochemical Journal*.

[67] Perez-Velazquez M., Maldonado-Othón C. A., González-Félix M. L. (2024). Molecular Weights and Optimum Temperature and pH for Pepsin Activity of Three Sciaenid Finfish Species From the Gulf of California. *Archives of Biological Sciences*.

[68] Wang G., Jiang Y., Jiang J., Chen L. (2021). Effects of Feed Supplemented With Probiotics on the Culture of Sea Cucumber *Apostichopus japonicus*. *Aquaculture Nutrition*.

[69] Pandiyan P., Balaraman D., Thirunavukkarasu R. (2013). Probiotics in Aquaculture. *Drug Invention Today*.

[70] Wang J.-H., Zhao L.-Q., Liu J.-F., Wang H., Xiao S. (2015). Effect of Potential Probiotic *Rhodotorula benthica* D30 on the Growth Performance, Digestive Enzyme Activity and Immunity in Juvenile Sea Cucumber *Apostichopus japonicus*. *Fish & Shellfish Immunology*.

[71] Liao M., Ren T., He L., Jiang Z., Han Y. (2014). Optimum Dietary Protein Level for Growth and Coelomic Fluid Non-Specific Immune Enzymes of Sea Cucumber *Apostichopus japonicus* Juvenile. *Aquaculture Nutrition*.

[72] Peres H., Oliva-Teles A. (2009). The Optimum Dietary Essential Amino Acid Profile for Gilthead Seabream (*Sparus aurata*) Juveniles. *Aquaculture*.

[73] Rodrigues A. T., Mansano C. F., Khan K. U. (2020). Ideal Profile of Essential Amino Acids for Nile Tilapia (*Oreochromis niloticus*) in the Finishing Growth Phase. *Aquaculture Research*.

[74] Eliseikina M. G., Magarlamov T. Y. (2002). Coelomocyte Morphology in the Holothurians *Apostichopus japonicus* (Aspidochirota: Stichopodidae) and *Cucumaria japonica* (Dendrochirota: Cucumariidae). *Russian Journal of Marine Biology*.

[75] Kudriavtsev I. V., Polevshchikov A. V. (2004). Comparative Immunological Analysis of Echinoderm Cellular and Humoral Defense Factors. *Zhurnal Obshcheĭ Biologii*.

[76] Zhong L., Hu Y., Hu Y. (2019). Effects of Dietary Tea Polyphenols on Growth, Immunity and Lipid Metabolism of Juvenile Black Carp *Mylopharyngodon piceus*. *Aquaculture Research*.

[77] Ahmadi A., Bagheri D., Hoseinifar S. H., Morshedi V., Paolucci M. (2022). Beneficial Role of Polyphenols as Feed Additives on Growth Performances, Immune Response and Antioxidant Status of *Lates Calcarifer* (Bloch, 1790) Juveniles. *Aquaculture*.

[78] Mi J., Liu D., Qin C. (2023). Dietary (−)-Epicatechin Supplementation Regulates Myofiber Development, Fillet Quality, and Antioxidant Status of Yellow River Carp (*Cyprinus carpio*). *Aquaculture*.

[79] Li Y., Liao K., Ji R. (2019). Effects of Tea Polyphenols on Growth, Antioxidant Capacity and Lipid Metabolism Related Genes Expression of Turbot (*Scophthalmus maximus*). *Journal of Fisheries of China*.

[80] Rabinkov A., Miron T., Konstantinovski L., Wilchek M., Mirelman D., Weiner L. (1998). The Mode of Action of Allicin: Trapping of Radicals and Interaction With Thiol Containing Proteins. *Biochimica et Biophysica Acta (BBA) - General Subjects*.

[81] Li Y., Wang Y., Zhang W. (2023). Effects of Different Kinds of Compound Attractant in Plant Protein Diet on Growth, Immune and Intestinal Digestive Enzyme Activities of Genetically Improved Farmed Tilapia (*Oreochromis niloticus*). *Chinese Journal of Animal Nutrition*.

[82] Huang Z., Aweya J. J., Zhu C. (2020). Modulation of Crustacean Innate Immune Response by Amino Acids and Their Metabolites: Inferences From Other Species. *Frontiers in Immunology*.

[83] Gao T., Li Y., Zhang J., Liu Y., Zhou B. (2012). The Effect of Protein on Growth, Digestion and Immunity of Turbot (*Scophthatmus maximus* L.) in Industrial Culture. *Marine Sciences*.

[84] Li X. (2013). *Researches on Selecting Feedstuff and Dietary Protein Requirement of Juvenil Apostichopus japonicus*.

[85] Bao P. (2019). *Studies on the Nutritive Value of Commonly Used Feed Ingredients for Sea Cucumber (Apostichopus japonicus) and the Efficient Use of Major Nutrients in the Feeds*.

